# Our Circulation

**Published:** 1884-07

**Authors:** John S. Prather

**Affiliations:** Business Assistant, Jas. P. Harrison & Co.


### OUR CIRCULATION.

I hereby certify that we print four thousand copies of THE ATLANTA MEDICAL AND SURGICAL JOURNAL for July.

JOHN S. PRATHER,
*Business Assistant, Jas. P. Harrison & Co.*

Our circulation has increased rapidly since March 1st, and to satisfy any doubting Thomas, we publish the above certificate as to the actual number printed, from the Business Manager of the publishing house.

